# The Water Cure, or Hydropathy

**Published:** 1847-02

**Authors:** 


### EDITORIAL DEPARTMENT.

*The Water Cure, or Hydropathy. From the British and Foreign Medical Review.* By JOHN FORBES, M. D., F. R. S., *Editor of British and Foreign Medical Review, &c.* Philadelphia, Lindsay & Blakeston, 1847, 12 mo. pp. 92.

This may not inaptly be called a sequel to the Review of Homœopathy by the same writer, which gave rise to such severity of criticism that the author felt it incumbent on him to make some apologetical explanations. The present article is written in a similar vein of disparagement of legitimate medicine, and excessive indulgence toward Quackery. Dr. Forbes seems resolutely bent on being considered peculiarly tolerant and independent; and the better to maintain this character, he makes, at the outset, an assumption entirely unwarrantable—certainly unwarrantable as regards the profession of this country, and, we cannot but think, equally so as regards the profession in Great Britain. He says Hydropathy is a "tabooed subject, being entirely excluded from medical journals, and medical books, or only admitted into them for the purpose of being ridiculed or utterly denounced," &c. "But," he continues, "we have been too long accustomed to speak our opinions openly and boldly, when we believed them to be just, whether they were in accordance with the current notions or not, to be deterred, on the present occasion, by any apprehended risk of offending mere professional conventionalism," &c. We should say this were a grave charge against the Medical profession, if it were not supremely ridiculous. Does Dr. Forbes really think so meanly of the profession, as to suppose that were he to offer any observations respecting the therapeutical efficacy of water, that they would not be received with proper consideration, because, forsooth, some persons believe, or affect to believe, that its efficacy is extensive and indiscriminate enough for it to be considered a panacea, or cure for all diseases? We cannot but suspect this arrogation of a degree of candor and boldness so far above the common professional standard, is intended particularly for unprofessional readers, a large body of whom are obstinately determined that medical men must, from principle, oppose every thing which conflicts with old-established opinions, and like the Jews of old, that they cannot believe any good to come out of Nazareth!

Dr. Forbes professes to bestow an elaborate investigation upon the success of Hydropathy—but whence does he derive the facts by which to judge of the merits of the water cure? He declares that he enters upon the investigation of this system "as propounded and practiced by Preissnitz and his disciples." He admits that the medical profession have, "naturally enough, and not inexcusably, treated it with contempt," from its being, "for the most part, adopted and professed by lay-practitioners, and by medical men of somewhat equivocal reputation." He also regrets that "there is, and has been from the beginning, not a little quackery and mystification mixed up with really effective practice in hydropathic proceedings."

He, also, considers it necessary to say, that he shall "make no apology to our authors for having, on several occasions, refused their evidence and rejected their conclusions." Yet, after this full admission of the disqualifications of the disciples of the water cure, and of his want of confidence in their good faith, he invites our attention to an investigation of which he distinctly avows the materials to have been derived mainly from the published writings of Hydropathists!

And what is the general conclusion at which he would have us arive? Why, simply this: "that cold water, applied in the manner of the hydropathists, is a powerful modifier of the human body, both in health and disease; and, when weighed in the therapeutic balance, with other medicines, merits, at least, a fair trial in legitimate practice."

Surely this is a most "lame and impotent conclusion," after such declamation about "freely and fearlessly promulgating, (his opinions) careless of personal consequences."

The truth is, had Dr. Forbes communicated the results of his own observations on the remedial effects of the water-cure, or had he reported the observations of others in whose competency and honesty he himself had confidence, there can be no doubt as to the reception which they would have met with from the profession.—We happen to know that this system has to some extent been applied in one of the best regulated hospitals of this country; and if the results should be deemed sufficiently important to be communicated, we do not imagine that the gentlemen who have conducted the observations have any reason to anticipate ridicule and contempt. We speak only in behalf of the profession of this country; it may be otherwise in England, and, if so, we are ready to go with Prof. Paine in the belief that the condition of American Medicine is far in the ascendant when compared with its condition in the British Islands. It is not only unjust, but insulting to the profession of this country to assert that any subject is *tabooed.* In one of our oldest and most respectable Medical Periodicals, Homœopathy itself has been permitted to present its claims, nor do we suppose that any Journal in the country would deny admission to authenticated reports from any correspondent whose scientific and moral character is unexceptionable, whatever might be their bearing upon the subjects of the popular delusions of the day.

But Dr. Forbes does not profess to have accumulated either by personal investigations, or authenticatod reports, satisfactory data for an examination of the merits of Hydropathy. He candidly admits that the evidence upon which he reasons is not good evidence. We say, then, that as a medical philosopher he was bound to have deferred his review until he was in possession of sufficient, reliable materials. Admit the logical propriety of accepting the statements of the originators and followers of new systems, as adequate data for reasoning upon the merits of such systems, and where would it not lead us! Every pretended panacea would be abundantly sustained by the numerous certificates and affidavits with which their virtues are heralded to the Public! If with the greatest circumspection false facts are the bane of medical science, what would be the condition of things if this degree of latitude were allowable!

Judging from the tenor of the review, we cannot resist the impression, that it was written with an eye to the approbation of certain unprofessional readers. Dr. F.'s motives are best known to himself. It is not difficult to divine what these may have been, but we are not disposed to speculate on that subject. At all events, we are bound to believe that Dr. F. has too much penetration and critical acumen not to have perceived what would be the popular effect of such an article, and what the general estimation of it by the medical profession. He cannot be surprised at its being circulated, as a defense of Hydropathy by a distinguished medical editor, and that his name and reputation will be cited as being fully committed to the extravagances of this system, by hundreds who will never read the article. He cannot but have perceived, that while, as a philosophical examination of the subject, it amounts to nothing, (which he virtually admits) its general spirit and tendency are such that the profession will feel aggrieved, and empiricism will derive countenance and support so far as the production exerts any influence upon the public mind. It is unreasonable to suppose that he has not voluntarily chosen thus to compromise, in a measure, what is due to his own professional character, and to the character of medical science in this publication, holding, at the same time, the position of editor of a medical journal which advertises that "no terms are kept with quacks or quackery."

